# Association of the composite dietary antioxidant index with advanced cardiovascular–kidney–metabolic syndrome in U.S. adults: A cross-sectional study based on NHANES 2001–2018

**DOI:** 10.1097/MD.0000000000050849

**Published:** 2026-09-25

**Authors:** Yubin Wang, Yingxuan Huang, Chanchan Lin, Xinqi Chen, Yingyi Li, Yisen Huang, Xiaoqiang Liu

**Affiliations:** a Department of Gastroenterology, First Hospital of Quanzhou Affiliated to Fujian Medical University, Quanzhou, Fujian, China.

**Keywords:** advanced CKM, CKM staging, composite dietary antioxidant index, NHANES, oxidative stress

## Abstract

This study aimed to examine the association between the composite dietary antioxidant index (CDAI) and advanced cardiovascular–kidney–metabolic syndrome (CKM) and characterize its dose–response shape. This cross-sectional study included 23,968 National Health and Nutrition Examination Survey participants aged ≥20 years (2001–2018). Advanced CKM was stages 3 and 4 combined, per the 2023 American Heart Association framework. The CDAI was the sum of sex-specific z-scores for 6 dietary antioxidants (vitamins A, C, and E, carotenoids, zinc, and selenium) from a single 24-hour recall, analyzed in quartiles (Q1–Q4). Multivariable logistic regression assessed the association, and restricted cubic splines tested nonlinearity. Multiple imputation, propensity score matching, survey-weighted models, and subgroup analyses evaluated robustness. Crude prevalence of advanced CKM fell from 33.6% (Q1) to 20.8% (Q4). After full adjustment, odds ratios (ORs) vs Q1 were 0.87 (95% confidence interval 0.78–0.97) for Q2, 0.79 (0.70–0.89) for Q3, and 0.83 (0.72–0.95) for Q4 (*P* for trend = .002). Splines showed an L-shaped association with a nadir at a CDAI of approximately 0.36; below this threshold each 1-unit increase was associated with marginally lower odds (OR 0.96, 95% confidence interval 0.92–1.00, *P* = .048), whereas above it the association plateaued. The associations were consistent across age, sex, smoking, alcohol intake, poverty income ratio, and physical activity strata (all *P* for interaction > .05). After multiple imputation the Q4 OR was 0.77 (0.69–0.85); propensity score–matched and survey-weighted estimates were directionally consistent, with a significant trend in each (*P* for trend = .045, .039). A higher food-derived CDAI was independently associated with a lower prevalence of advanced CKM, with the inverse association confined to the low-to-moderate intake range. These findings are consistent with dietary guidance emphasizing whole foods rich in diverse antioxidants, although the cross-sectional design precludes causal inference.

## 1. Introduction

Cardiovascular–kidney–metabolic syndrome (CKM) is a novel staging framework proposed by the American Heart Association in 2023 to integrate obesity, type 2 diabetes, chronic kidney disease (CKD), and atherosclerotic cardiovascular disease (ASCVD) into an interrelated pathological network.^[1]^ Based on the 2011 to 2020 National Health and Nutrition Examination Survey (NHANES) data, nearly 90% of U.S. adults are in CKM stages 1 to 4, and approximately 15% are in the advanced stages 3 or 4, indicating a substantial public health burden.^[2]^ Individuals in stages 3 and 4, collectively termed advanced CKM, are characterized by clinical ASCVD and/or very high-risk CKD and have substantially higher risks of mortality and rehospitalization, underscoring the need for modifiable lifestyle interventions to slow disease progression.^[3,4]^

Oxidative stress is recognized as a common pathway in heart–kidney–metabolic dysregulation; excess reactive oxygen species (ROS) accelerate low-density lipoprotein oxidation, inhibit endothelial nitric oxide production, and trigger inflammatory–fibrotic injury in the myocardium, glomeruli, and pancreatic β-cells.^[5–7]^ CKD and diabetes amplify the oxidative burden through chronic inflammation, creating a vicious cycle.^[8,9]^ Therefore, sustained intake of natural dietary antioxidants is a potential strategy for mitigating CKM cascade damage.^[10,11]^ Compared with single-nutrient analysis, the Composite Dietary Antioxidant Index (CDAI), the standardized sum of the intake of vitamins A, C, and E, carotenoids, zinc, and selenium, captures the overall antioxidant capacity more comprehensively.^[12]^ Cross-sectional and cohort studies have linked a higher CDAI to a lower 10-year ASCVD risk^[13]^ and have reported inverse associations with CKD and cardiovascular mortality, although the shape of the dose–response relationship has varied across studies. Nevertheless, these studies focused on single-organ endpoints and did not examine CDAI against a system-level outcome, such as advanced CKM.

Prior studies have shown that an oxidative balance score, a composite of pro- and antioxidant exposures, tracks CKM staging and mortality risk, and that the triglyceride-glucose index, a surrogate of insulin resistance, is related to cardiometabolic risk relevant to CKM.^[14,15]^ However, neither composite isolates food-derived antioxidant intake from lifestyle and pro-oxidant components, and the shape of their association with CKM stage has not been characterized.

Given that the CKM framework offers an integrative perspective for chronic disease management and that the CDAI is a practical marker of a modifiable exposure, we used the large NHANES 2001 to 2018 sample to examine whether dietary antioxidant intake is associated with the advanced stages of CKM syndrome, and to characterize the shape of that association across the full range of intake. We hypothesized that a higher CDAI would be inversely associated with advanced CKM prevalence, independent of confounders, and that a dose threshold may exist. Elucidating this relationship could fill a gap in CKM prevention evidence and provide quantitative support for precision nutrition strategies driven by diverse, antioxidant-rich diets.

## 2. Methods

### 2.1. Study design and data source

This cross-sectional analysis used data from the NHANES, which employs a multistage stratified probability design to collect nationally representative health and nutrition data from U.S. residents. The present study utilized the 2001 to 2018 survey cycles. The NHANES protocols were approved by the National Center for Health Statistics ethics review board, and all participants provided written informed consent. This study adhered to the Strengthening the Reporting of Observational Studies in Epidemiology guidelines for observational studies.

### 2.2. Study population and eligibility

Among 55,081 NHANES participants aged ≥20 years (2001–2018), 10,580 participants lacking dietary data required for CDAI calculation were excluded, leaving 44,501 participants. Subsequently, 15,565 participants without CKM classification indicators (estimated glomerular filtration rate [eGFR], urinary albumin-to-creatinine ratio, or ASCVD records) were removed, and 4968 were excluded due to missing covariates or pregnancy (4474 with incomplete data; 494 pregnant). A total of 23,968 adults with complete information were included (Fig. 1).

**Figure 1.**
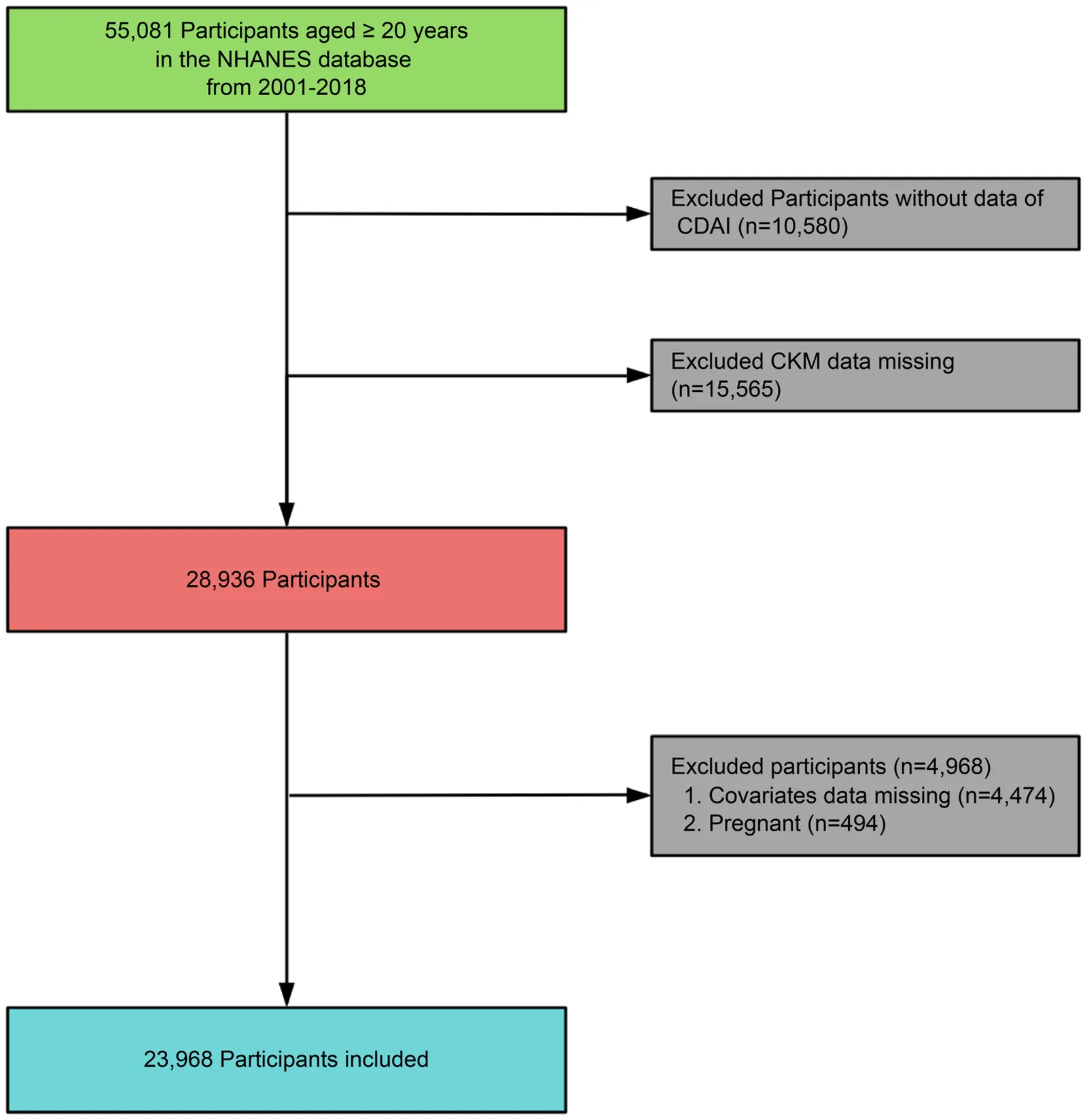
Flow chart of the screening of the NHANES 2001 to 2018 participants. CDAI = composite dietary antioxidant index, CKM = cardiovascular–kidney–metabolic.

### 2.3. Definition of the composite dietary antioxidant index (CDAI)

The NHANES dietary interview “What We Eat in America” records participants’ food and nutrient intake, with nutrient values assigned using the United States Department of Agriculture Food and Nutrient Database for Dietary Studies. Participants were invited to complete 2 24-hour dietary recalls: the first was collected in the Mobile Examination Center, and the second was obtained by telephone 3 to 10 days later. Only the first recall was included in this analysis. The CDAI was computed according to established methods.^[16]^ In this analysis, nutrient contributions from dietary supplements, antacids, and other medications were excluded. Intakes of 6 dietary antioxidants (zinc, selenium, preformed vitamin A [retinol], vitamin C, vitamin E, and total carotenoids) were standardized within sex to z-scores by subtracting the sex-specific mean and dividing by the corresponding standard deviation. The CDAI was calculated as the sum of the 6 standardized values.


$$CDAI=\overset{n=6}{\underset{i=1}{\sum }}\frac{each\;intake-mean}{SD}$$

### 2.4. Definition of advanced CKM

The CKM staging system classifies adult heart, kidney, and metabolic health into stages 0 to 4: stage 0 indicates ideal CKM health; stage 1 denotes general or abdominal obesity or adiposopathy without metabolic or kidney abnormalities; stage 2 involves ≥1 metabolic risk factor (e.g., diabetes, hypertension) or early CKD (eGFR < 60 mL/minutes/1.73 m^2^ or urinary albumin-to-creatinine ratio ≥30 mg·g^−1^) but no cardiovascular organ damage; stage 3 represents subclinical or clinical ASCVD or high-risk CKD (eGFR < 45 mL/minutes/1.73 m^2^), usually accompanied by ≥1 metabolic phenotype; and stage 4 comprises clinical ASCVD (myocardial infarction, stroke, peripheral artery disease, or heart failure) often with advanced CKD or end-stage kidney disease.^[17]^ A comprehensive description of the staging criteria is provided in Supplementary Table 1, Supplemental Digital Content 1.

The clinical conditions used to assign CKM stage were operationalized as follows. Cardiovascular disease (CVD) was defined by a self-reported physician diagnosis of coronary heart disease, angina, myocardial infarction, heart failure, or stroke.^[18]^ Hypertension was defined as a mean systolic blood pressure ≥140 mm Hg, a mean diastolic blood pressure ≥90 mm Hg, a self-reported physician diagnosis, or the use of antihypertensive medication.^[19]^ Dyslipidemia was defined as the use of lipid-lowering therapy, triglycerides ≥150 mg/dL, total cholesterol ≥200 mg/dL, low-density lipoprotein cholesterol ≥130 mg/dL, or high-density lipoprotein cholesterol <40 mg/dL.^[20]^ Diabetes was defined as a physician diagnosis, glycated hemoglobin ≥6.5%, fasting plasma glucose ≥7.0 mmol/L, a 2-hour oral glucose tolerance test or random plasma glucose ≥11.1 mmol/L, or the use of glucose-lowering medication.^[21]^ Because these conditions constitute the staging criteria, they were not included as adjustment covariates. Because stages 3 to 4 indicate the highest risk due to combined organ damage and metabolic abnormalities, they were merged as advanced CKM, the main outcome; participants at stages 0 to 2 were classified as without advanced CKM and served as the reference group.^[22]^

### 2.5. Covariate assessment

Covariates were selected based on the literature and clinical knowledge and operationalized according to the NHANES data dictionary.^[23–25]^ Demographics: age (years), sex (male/female), and race (non-Hispanic White, non-Hispanic Black, Mexican American, and other). Socioeconomic status: marital status (married/living with partner, never married/other), poverty income ratio (PIR; 1.00–1.30, 1.31–3.50, > 3.50), and education level (less than high school, high school or equivalent, above high school).^[26]^ Lifestyle: total energy intake (kcal/day), (body mass index, kg/m^2^), smoking status (never <100 cigarettes, former ≥100 but not current, current ≥100 and currently smoking),^[27]^ alcohol intake (lifetime <12 drinks, former ≥12 drinks but none in the past year, current ≥12 drinks in the past year),^[28]^ and weekly physical activity (metabolic equivalent of task-min/week; inactive 0, insufficient 1 to 599, sufficient ≥600).^[29]^ CVD, hypertension, dyslipidemia, and diabetes are defined under the CKM staging criteria above; because they form part of the outcome definition, adjusting for them would introduce over adjustment bias, and they were therefore excluded from the covariate set.

### 2.6. Statistical analysis

All analyses used R 4.3.2 and Free Statistics 2.1.1. A two-sided *P* < .05 was considered statistically significant. Continuous variables are expressed as mean ± standard deviation and were compared using analysis of variance; categorical variables are n (%) and were compared using the *χ*^2^ test. CDAI was entered into multivariable logistic models as both continuous and quartile variables. Covariates were chosen based on clinical relevance, univariable *P* < .05, or a >10% change in effect estimate. Three models were built: crude (unadjusted); Model 1 (adjusted for age and sex); and Model 2 (Model 1 plus race, marital status, PIR, education, body mass index, total energy intake, smoking status, alcohol intake, and physical activity). Trends across quartiles were tested by assigning the median value of each quartile and modeling it as a continuous variable. Nonlinearity was assessed using restricted cubic splines (knots at the 5th, 35th, 65th, and 95th percentiles) in Model 2. When nonlinearity was present (*P* < .05), the inflection point was defined as the spline nadir (minimum predicted log-odds), after which we fitted a 2-piecewise logistic regression with the breakpoint fixed at that value. Effect heterogeneity was examined by adding interaction terms for age, sex, smoking, alcohol intake, PIR, and physical activity within the analytic sample (n = 23,968).

Although NHANES is probability-based, our analytic sample excluded participants with missing dietary data, CKM indicators, or covariates, as well as pregnant individuals; if these exclusions were nonrandom, selection bias could affect the generalizability or magnitude of the association between CDAI and CKM. To address this a priori, we prespecified robustness analyses comprising multiple imputation by chained equations (*m* = 5) for participants with at least 1 missing covariate (n = 4474), with CDAI quartile and the outcome included as predictors only (not imputed), continuous variables imputed via predictive mean matching and categorical variables via logistic regression, and estimates combined using Rubin’s rules; 1:1 nearest-neighbor propensity score matching (caliper 0.02 on the logit, without replacement) comparing Q2, Q3, and Q4 each with Q1, with propensity scores estimated from all Model 2 covariates and covariate balance assessed using (standardized mean differences <0.10); and survey-weighted logistic regression that accounted for the NHANES complex design (strata SDMVSTRA, PSUs SDMVPSU) and Day-1 dietary weights (WTDRD1), with 2-year weights pooled across 2001 to 2018 by dividing by 9. These steps mitigate but cannot eliminate potential sampling and selection biases.

## 3. Results

### 3.1. Baseline characteristics

As shown in Table 1, among 23,968 U.S. adults (NHANES 2001–2018), CDAI ranged from − 7.1 to 77.9 and was stratified into quartiles. Higher CDAI was associated with younger age (mean, 54.3 years in Q1 [lowest CDAI] vs 50.6 years in Q4 [highest CDAI]), better socioeconomic indicators (education, income), healthier lifestyles (physical activity), and stepwise declines in CVD, diabetes, and hypertension. The crude prevalence of advanced CKM likewise decreased stepwise across ascending quartiles, from 33.6% in Q1 to 20.8% in Q4. The proportion of current alcohol users increased across the CDAI quartiles and was highest in Q4.

**Table 1:** Initial characteristics of individuals according to CDAI quartiles.

Variables	Total	Q1 (-7.1, -2.3)	Q2 (-2.2, -0.4)	Q3 (-0.3, 2.0)	Q4 (2.1, 77.9)	*P* value
Number of participants	23,968	5928	5893	5976	6171	
Age, Mean ± SD	52.8 ± 17.5	54.3 ± 17.8	53.9 ± 17.6	52.6 ± 17.5	50.6 ± 17.0	<.001
Sex, n (%)						<.001
Male	12,267 (51.2)	2835 (47.8)	3076 (52.2)	3142 (52.6)	3214 (52.1)	
Female	11,701 (48.8)	3093 (52.2)	2817 (47.8)	2834 (47.4)	2957 (47.9)	
Race, n (%)						<.001
Non-Hispanic White	10,963 (45.7)	2416 (40.8)	2770 (47.0)	2883 (48.2)	2894 (46.9)	
Non-Hispanic Black	5097 (21.3)	1547 (26.1)	1204 (20.4)	1130 (18.9)	1216 (19.7)	
Mexican American	3955 (16.5)	979 (16.5)	987 (16.7)	989 (16.5)	1000 (16.2)	
Other race	3953 (16.5)	986 (16.6)	932 (15.8)	974 (16.3)	1061 (17.2)	
Marital status, n (%)						<.001
Married/living with partner	14,547 (60.7)	3282 (55.4)	3633 (61.6)	3740 (62.6)	3892 (63.1)	
Never married/other	9421 (39.3)	2646 (44.6)	2260 (38.4)	2236 (37.4)	2279 (36.9)	
PIR, n (%)						<.001
1–1.3	7276 (30.4)	2284 (38.5)	1811 (30.7)	1591 (26.6)	1590 (25.8)	
1.31–3.50	9328 (38.9)	2369 (40.0)	2340 (39.7)	2379 (39.8)	2240 (36.3)	
>3.50	7364 (30.7)	1275 (21.5)	1742 (29.6)	2006 (33.6)	2341 (37.9)	
Education level, n (%)						<.001
Less than high school	6317 (26.4)	2100 (35.4)	1574 (26.7)	1418 (23.7)	1225 (19.9)	
High school or equivalent	5580 (23.3)	1500 (25.3)	1449 (24.6)	1302 (21.8)	1329 (21.5)	
Above high school	12,071 (50.4)	2328 (39.3)	2870 (48.7)	3256 (54.5)	3617 (58.6)	
BMI, kg/m^2^, Mean ± SD	29.6 ± 6.9	29.6 ± 6.9	29.8 ± 6.8	29.7 ± 6.9	29.5 ± 7.2	.194
Total energy intake, Mean ± SD	2074.6 ± 970.5	1322.2 ± 527.2	1846.0 ± 596.1	2237.6 ± 717.6	2857.9 ± 1149.0	<.001
Alcohol intake, n (%)						<.001
Never	3398 (14.2)	1006 (17.0)	795 (13.5)	834 (14.0)	763 (12.4)	
Former	4713 (19.7)	1384 (23.3)	1233 (20.9)	1066 (17.8)	1030 (16.7)	
Current	15,857 (66.2)	3538 (59.7)	3865 (65.6)	4076 (68.2)	4378 (70.9)	
Smoking status, n (%)						<.001
Never	12,451 (51.9)	2828 (47.7)	2979 (50.6)	3182 (53.2)	3462 (56.1)	
Former	6647 (27.7)	1567 (26.4)	1722 (29.2)	1717 (28.7)	1641 (26.6)	
Current	4870 (20.3)	1533 (25.9)	1192 (20.2)	1077 (18.0)	1068 (17.3)	
Physical activity, n (%)						<.001
Inactive	7097 (29.6)	2154 (36.3)	1849 (31.4)	1599 (26.8)	1495 (24.2)	
Insufficiently active	10,579 (44.1)	2399 (40.5)	2615 (44.4)	2748 (46.0)	2817 (45.6)	
Sufficiently active	6292 (26.3)	1375 (23.2)	1429 (24.2)	1629 (27.3)	1859 (30.1)	
CVD, n (%)						<.001
No	20,059 (83.7)	4696 (79.2)	4841 (82.1)	5116 (85.6)	5406 (87.6)	
Yes	3909 (16.3)	1232 (20.8)	1052 (17.9)	860 (14.4)	765 (12.4)	
Hyperlipidemia, n (%)						<.001
No	5762 (24.0)	1314 (22.2)	1325 (22.5)	1473 (24.6)	1650 (26.7)	
Yes	18,206 (76.0)	4614 (77.8)	4568 (77.5)	4503 (75.4)	4521 (73.3)	
Hypertension, n (%)						<.001
No	12,122 (50.6)	2721 (45.9)	2870 (48.7)	3169 (53.0)	3362 (54.5)	
Yes	11,846 (49.4)	3207 (54.1)	3023 (51.3)	2807 (47.0)	2809 (45.5)	
Diabetes, n (%)						<.001
No	17,968 (75.0)	4260 (71.9)	4332 (73.5)	4568 (76.4)	4808 (77.9)	
Yes	6000 (25.0)	1668 (28.1)	1561 (26.5)	1408 (23.6)	1363 (22.1)	
Advanced CKM, n (%)						<.001
No	17,482 (72.9)	3937 (66.4)	4160 (70.6)	4495 (75.2)	4890 (79.2)	
Yes	6486 (27.1)	1991 (33.6)	1733 (29.4)	1481 (24.8)	1281 (20.8)	

BMI = body mass index, CDAI = composite dietary antioxidant index, CKM = cardiovascular–kidney–metabolic, CVD = cardiovascular disease, PIR = poverty income ratio.

### 3.2. Association between CDAI and advanced CKM

As shown in Table 2, per 1-unit CDAI increase, the odds of advanced CKM were 6% lower in the crude model (odds ratio [OR] 0.94, 95% confidence interval [CI] 0.93–0.95), and the estimate remained significant after adjusting for age and sex (OR 0.96, 95% CI 0.95–0.97), but was attenuated to the null in the fully adjusted model (OR 1.00, 95% CI 0.98–1.01). When CDAI was entered as quartiles (Q1 as the reference), the fully adjusted ORs (95% CIs) were 0.87 (0.78–0.97) for Q2, 0.79 (0.70–0.89) for Q3, and 0.83 (0.72–0.95) for Q4, with a significant trend across quartiles (*P* for trend = .002).

**Table 2:** Association between CDAI and advanced CKM syndrome.

	Crude model		Model 1		Model 2	
	OR (95%CI)	*P* value	OR (95%CI)	*P* value	OR (95%CI)	*P* value
Continuous CDAI	0.94 (0.93~0.95)	<.001	0.96 (0.95~0.97)	<.001	1 (0.98~1.01)	.643
CDAI quartile						
Q1	Reference		Reference		Reference	
Q2	0.82 (0.76~0.89)	<.001	0.75 (0.67~0.83)	<.001	0.87 (0.78~0.97)	.015
Q3	0.65 (0.60~0.71)	<.001	0.61 (0.55~0.68)	<.001	0.79 (0.70~0.89)	<.001
Q4	0.52 (0.48~0.56)	<.001	0.58 (0.52~0.65)	<.001	0.83 (0.72~0.95)	.008
* P* for trend		<.001		<.001		.002

The crude model was not adjusted for any covariates.

Model 1 was adjusted for age and sex.

Model 2 was further adjusted for race, marital status, PIR, education level, body mass index, total energy intake, smoking status, alcohol intake, and physical activity.

CDAI = composite dietary antioxidant index, CKM = cardiovascular–kidney–metabolic, PIR = poverty income ratio.

### 3.3. Dose–response relationship

The restricted cubic splines showed an L-shaped association (Fig. 2). Piecewise regression (Table 3) was consistent with this pattern: below the inflection point (CDAI = 0.36, which corresponds to the 58th percentile of the CDAI distribution in this sample and approximates the sample mean of 0.40), each 1-unit CDAI increase was associated with lower odds of advanced CKM (OR 0.96, 95% CI 0.92–1.00, *P* = .048), whereas no association was observed above the threshold (OR 1.01, 95% CI 0.98–1.04, *P* = .547).

**Table 3:** Threshold effect analysis of CDAI on advanced CKM syndrome.

CDAI	OR (95%CI)	*P* value
<0.36	0.96 (0.92~1.00)	.048
≥0.36	1.01 (0.98~1.04)	.547
Likelihood ratio test	–	<.001

The crude model was not adjusted for any covariates.

Model 1 was adjusted for age and sex.

Model 2 was further adjusted for race, marital status, PIR, education level, body mass index, total energy intake, smoking status, alcohol intake, and physical activity.

Estimates were derived from a 2-piecewise logistic regression fitted within Model 2, adjusted for age, sex, race, marital status, PIR, education level, body mass index, total energy intake, smoking status, alcohol intake, and physical activity. The upper limit of the 95% confidence interval for the CDAI < 0.36 segment is 0.9996, displayed as 1.00 after rounding to 2 decimal places.

CDAI = composite dietary antioxidant index, CKM = cardiovascular–kidney–metabolic, PIR = poverty income ratio.

**Figure 2.**
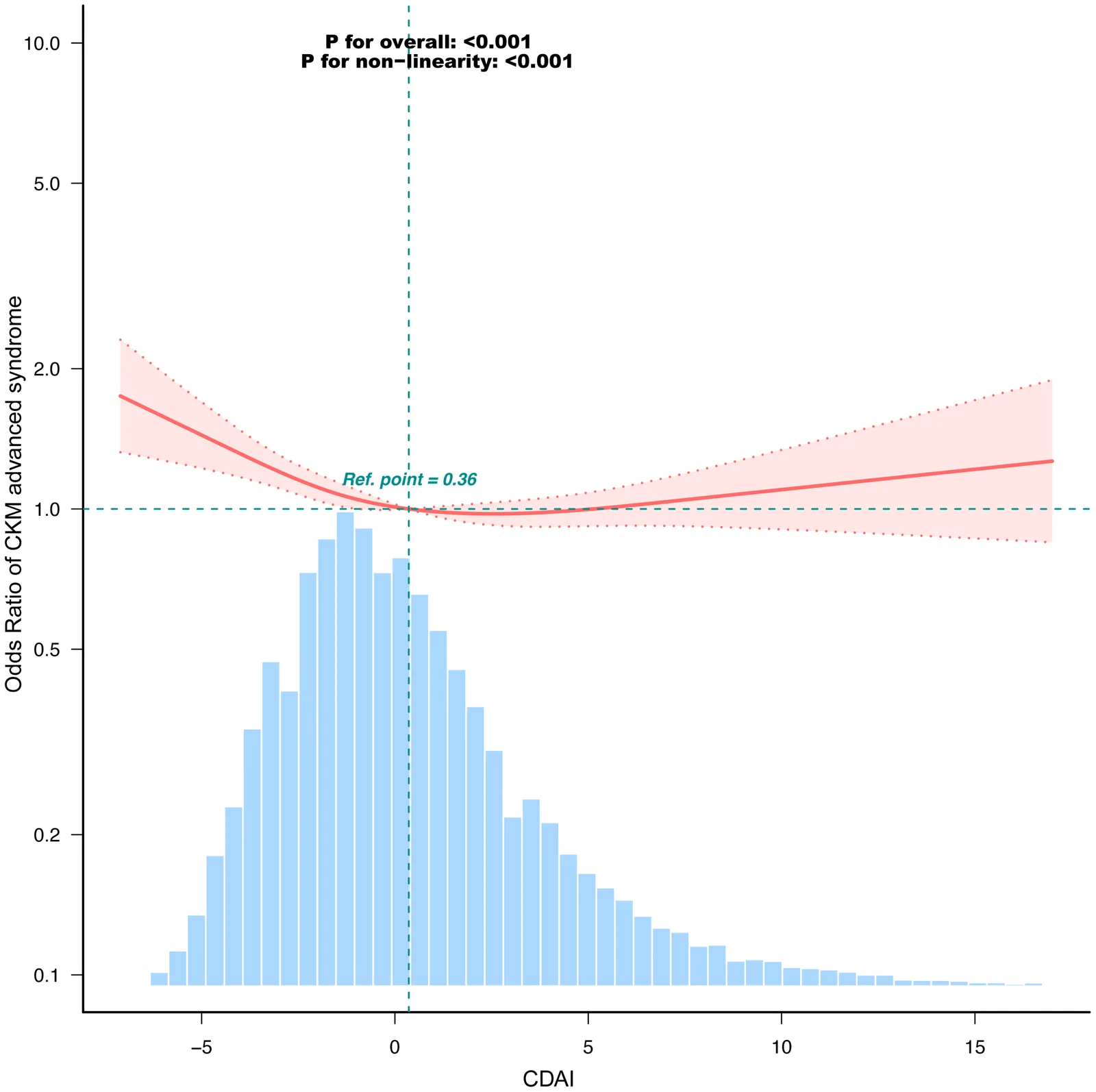
Analysis of the association between CDAI and advanced CKM syndrome using RCS. CDAI = composite dietary antioxidant index, CKM = cardiovascular–kidney–metabolic, PIR = poverty income ratio. Adjusted for age, sex, race, marital status, PIR = education level, BMI = total energy intake, smoking status, alcohol intake, and physical activity.

### 3.4. Subgroup and sensitivity analyses

Inverse associations were consistent across all 6 predefined subgroups, with no significant interactions (all *P* for interaction >.05; Fig. 3). After multiple imputation (n = 28,442; Supplementary Table 2, Supplemental Digital Content 2), the Q4 OR was 0.77 (95% CI 0.69–0.85). In propensity score–matched analyses (Supplementary Table 3, Supplemental Digital Content 3), the association was attenuated, and the contrast with Q1 remained significant for Q3 (OR 0.85, 95% CI 0.74–0.99, *P* = .034); the estimates for Q2 (OR 0.93, 95% CI 0.81–1.07) and Q4 (OR 0.86, 95% CI 0.73–1.02) were in the same direction but did not reach significance, and the trend across quartiles remained significant (*P* for trend = .045). In survey-weighted models that incorporated the NHANES complex design, the direction and trend were consistent. The per 1-unit estimate remained null (OR 1.00, 95% CI 0.98–1.02, *P* = .682), whereas the quartile contrasts showed a graded association versus Q1 (Q2 0.88, 95% CI 0.76–1.02; Q3 0.84, 95% CI 0.71–0.99; Q4 0.83, 95% CI 0.69–0.99), with a significant trend across quartiles (*P* for trend = .039); the Q2 contrast did not reach significance (Supplementary Table 4, Supplemental Digital Content 4).

**Figure 3.**
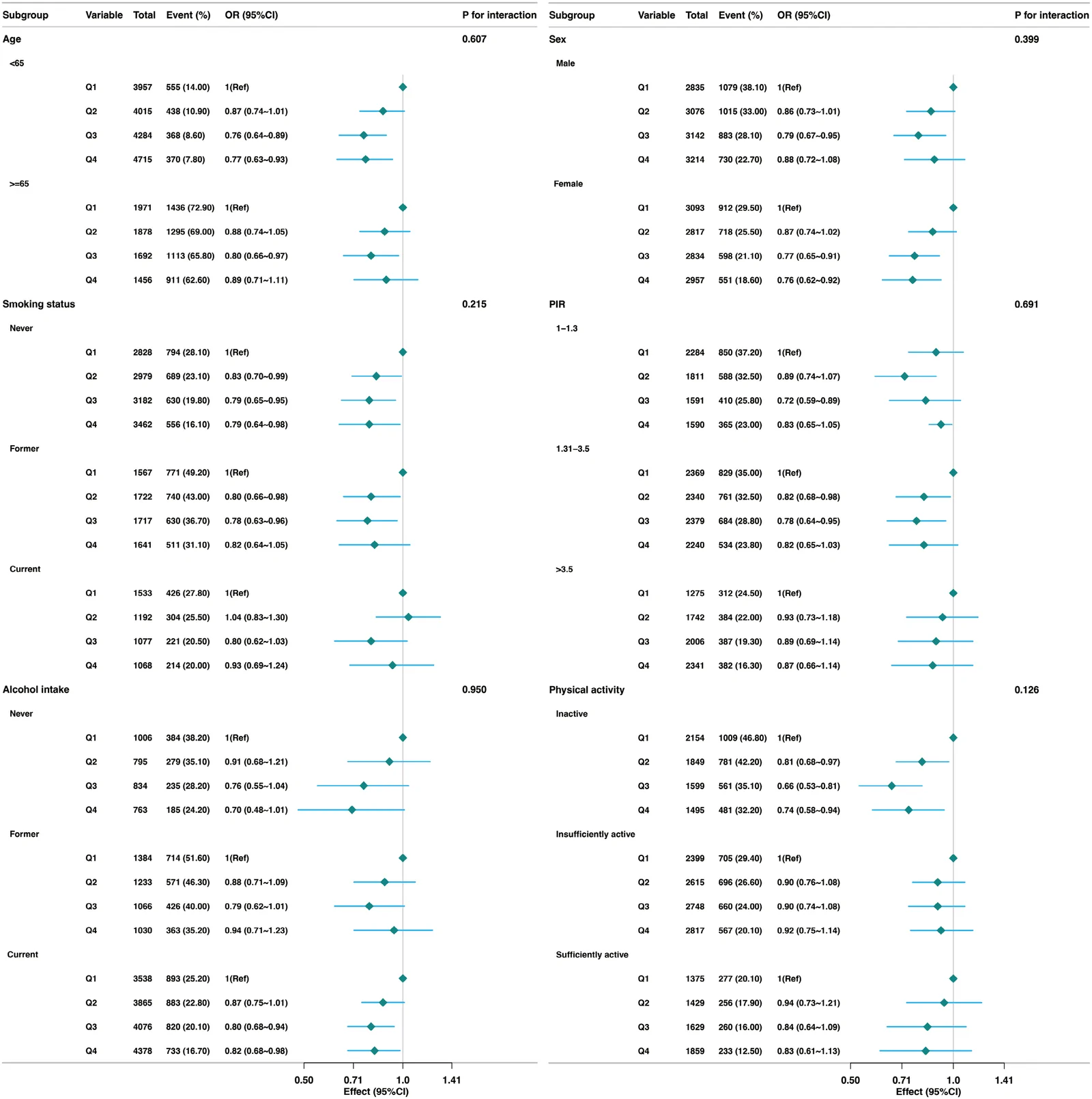
Subgroup analyses of the association between CDAI and advanced CKM syndrome. BMI = total energy intake, smoking status, alcohol intake, and physical activity, CDAI = composite dietary antioxidant index, CKM = cardiovascular–kidney–metabolic, PIR = poverty income ratio, Adjusted for age, sex, race, marital status, PIR = education level.

## 4. Discussion

This large cross-sectional analysis demonstrated that higher CDAI was independently and inversely associated with advanced CKM. Compared with the lowest quartile (Q1), Q2 to Q4 showed 13% to 21% lower odds of advanced CKM (*P* for trend = .002). The estimates were directionally consistent after multiple imputation, propensity score matching, and survey-weighted modeling, and the trend across quartiles remained significant in each of these analyses (*P* for trend < .001, .045, and .039, respectively). Restricted cubic splines showed an L-shaped dose–response relationship, with a nadir at a CDAI of approximately 0.36. Above this value the association plateaued, suggesting that the inverse association with advanced CKM was confined to the low-to-moderate range of antioxidant intake rather than strengthening progressively at higher intakes.

Most evidence on CDAI–health associations remains organ-specific. In cardiovascular research, the highest CDAI quartile has been associated with a 29% lower prevalence of CVD (OR 0.71, 95% CI 0.59–0.85),^[30]^ and with 23% lower all-cause mortality among adults with hypertension over a mean follow-up of 8.5 years (hazard ratio 0.77, 95% CI 0.68–0.87), although the corresponding association with cardiovascular mortality did not reach statistical significance (hazard ratio 0.83, 95% CI 0.67–1.04).^[31]^ Participants with heart failure have likewise been reported to have lower mean CDAI than those without.^[32]^ Reports of the dose–response shape, however, have been inconsistent: an L-shaped association has been described for ASCVD risk in postmenopausal women,^[33]^ whereas a linear inverse association was reported for all-cause and cardiovascular mortality.^[34]^ In kidney populations, the highest CDAI quartile was associated with a lower prevalence of CKD,^[35]^ and an L-shaped association with all-cause mortality has been reported among adults with CKD (*P* for nonlinearity =.032).^[36]^

Two features of this literature bear on our findings. First, the shape we observed is consistent with the L-shaped patterns reported for ASCVD and for mortality in CKD,^[33,36]^ but prior studies have almost uniformly reported curve shape without estimating where the curve flattens. Only 1 reported a numerical inflection point: a CDAI of 3.05 for CVD prevalence among women, below which each 1-unit increase was associated with 6% lower odds of CVD (OR 0.94, 95% CI 0.90–0.98).^[30]^ That value is higher than our nadir of 0.36, which is expected given the different outcome, the sex-restricted sample, and the exposure distribution. Because a threshold’s location depends on the exposure distribution and the assumed functional form, such values are not directly comparable across studies. To our knowledge, the present analysis is the first to estimate and report an explicit inflection point for a CDAI–CKM association. Second, associations with intermediate metabolic phenotypes have generally been described as linear or only weakly nonlinear, whereas the association we observed with a clinical, multi-organ endpoint was distinctly L-shaped. One interpretation is that graded differences persist across the intake range while dysfunction remains subclinical but saturate once established organ damage defines the outcome; this interpretation is hypothesis-generating and was not tested directly.

A smaller and more recent literature shares our outcome rather than our exposure. Composite oxidative balance measures have been related to CKM staging and mortality,^[24]^ and recent studies have examined nutrient intakes in relation to CKM stage and mortality and dietary patterns and micronutrients in relation to major adverse cardiovascular events among individuals in CKM stages 0 to 3.^[37,38]^ These studies differ from ours in 3 respects. They characterize the earlier segment of the CKM continuum, whereas we restricted attention to stages 3 and 4; they treat nutrients or dietary patterns individually, whereas the CDAI aggregates 6 antioxidants into a single standardized exposure; and none reports the shape of the exposure–outcome association across the full range of intake. The present study therefore addresses a segment of the continuum and a modeling question that this literature has not yet covered, rather than replicating it. The principal divergence from earlier CDAI reports concerns the continuous exposure term, which remained significant in several single-organ analyses but was null here after full adjustment. As set out below, we attribute this to the shape of the association across the exposure range rather than to an absence of association. It follows that per-unit estimates should not be compared across CDAI studies without reference to the exposure distribution and the assumed functional form.

Notably, the fully adjusted per-unit CDAI term was null, whereas the quartile contrasts and the trend test across quartiles remained significant, with the lowest odds observed in Q3 rather than Q4. This pattern is consistent with an L-shaped dose–response: a single linear coefficient for continuous CDAI averages the curve across the entire distribution, so that the steep decline at lower CDAI is offset by the plateau above approximately 0.36, yielding an overall slope near zero. Quartile modeling and the piecewise slopes, by contrast, isolate the low-to-moderate range in which the odds of advanced CKM are lower, and therefore detect significant differences. We also observed that the proportion of current alcohol users increased across the CDAI quartiles and was highest in Q4. Models adjusted for alcohol status and total energy intake produced materially unchanged estimates.

Several mechanisms may account for the inverse association observed at lower intakes. Fat-soluble antioxidants (vitamins A and E and carotenoids) and water-soluble vitamin C scavenge ROS, reduce low-density lipoprotein oxidation, and preserve endothelial nitric oxide synthase activity, attenuating atherogenesis.^[39,40]^ Selenium, as a constituent of glutathione peroxidase, and zinc, as a cofactor of Cu/Zn-superoxide dismutase, bolster endogenous defenses, limit mitochondrial dysfunction, and suppress inflammasome activation in the vasculature and kidney.^[40,41]^ Antioxidant-rich foods also supply polyphenols and fiber, modulating gut microbiota and short-chain fatty acid production, improving insulin sensitivity, and lowering systemic inflammation, which are key metabolic drivers of CKM progression.^[42]^ Finally, these compounds display synergy in bioavailability and redox potential; vitamins C and E regenerate each other’s active forms at physiological ratios, prolonging efficacy.^[43]^ Why the association did not strengthen further above the threshold is a separate question. Benefit may be bounded by endogenous redox signaling: moderate ROS fluctuations activate Nrf2 and upregulate endogenous defenses, whereas exogenous antioxidants that scavenge ROS before signal transduction blunt this adaptation.^[44]^ Antioxidant capacity appears itself to be dose-dependent, with moderate pro-reductive states producing adaptive cardiac remodeling in experimental models but hyper-reductive states producing pathological remodeling and reduced survival.^[45]^ Antagonism among components may also contribute, since the 6 nutrients are not absorbed independently of one another. This account fits the observed plateau but was not tested here and remains speculative.

Dietary antioxidant levels in the middle quartiles of the U.S. population may be associated with lower odds of advanced CKM. The information this adds to current practice is not a new intervention but a target and a stopping point. Because the association was concentrated below a CDAI of approximately 0.36, a value close to the sample mean and the 58th percentile of this population, the observed differences correspond to moving the lowest consumers toward average intake rather than to pushing intake into the upper tail. For adults already in CKM stages 3 and 4, who are typically under clinical follow-up and already receiving dietary advice, this argues for directing counseling effort toward those with the lowest antioxidant intake, and for favoring diversity of antioxidant-rich whole foods, such as colorful fruits and vegetables, nuts, legumes, and whole grains, over escalating single-nutrient supplementation. That emphasis is consistent with randomized evidence that high-dose vitamin E and beta-carotene supplementation provides no cardiovascular benefit and may increase all-cause mortality in general adult populations,^[46,47]^ and with the absence of demonstrated benefit from antioxidant therapy in adults with CKD,^[48]^ although no trial has directly compared these 2 strategies in CKM stages 3 and 4. Policymakers could integrate the CDAI into diet quality indices, such as the Healthy Eating Index, to make oxidative stress an actionable dimension of dietary assessment. We emphasize, however, that a cross-sectional association cannot establish that raising antioxidant intake alters CKM stage; these findings refine how existing guidance is targeted, and do not by themselves justify a new dietary prescription.

### 4.1. Strengths

Strengths include a large, nationally representative sample with socioeconomic diversity, construction of the CDAI from Day-1 24-hour dietary recalls, and the use of the 2023 American Heart Association CKM staging as a clinically relevant composite endpoint. Methodologically, we adjusted for extensive covariates, tested nonlinearity, applied multiple imputation, propensity score matching, and survey-weighted models, and evaluated effect modification, thereby enhancing robustness.

### 4.2. Limitations

Several limitations should be considered when interpreting these findings. First, the analysis was cross-sectional, so causal direction cannot be established and reverse causation remains possible: individuals with latent CKM deterioration may have modified their diets before the survey. Such behavioral change could attenuate the inverse association, if participants with more advanced disease increased their intake of antioxidant-rich foods after counseling, or exaggerate it, if clinical restrictions reduced these foods. Estimates should therefore be read as associations rather than effects. Second, the analytic sample excluded 15,565 participants who lacked CKM staging indicators; if these individuals differed systematically from those retained, selection bias and restricted generalizability are possible, and although multiple imputation, propensity score matching, and survey-weighted models yielded directionally consistent results, these procedures mitigate rather than eliminate such bias. Third, dietary exposure was derived from a single Day-1 24-hour recall, which is subject to misreporting and does not capture habitual intake; the resulting within-person variability introduces nondifferential exposure misclassification that would bias estimates toward the null and may have led us to underestimate the steepness of the association below the inflection point. Fourth, antioxidant intake from supplements was excluded by design, so total antioxidant exposure is underestimated among supplement users, and the CDAI reported here reflects food-derived intake only. Fifth, alcohol was adjusted for as a status variable, without information on amount, frequency, or beverage type, leaving residual confounding by drinking pattern possible. Finally, unmeasured confounding cannot be excluded; environmental toxicant exposure, psychosocial stress, and the pharmacological profiles of concomitant medications were not available.

### 4.3. Knowledge gaps

Several questions relevant to this association remain unanswered in the existing literature. The first concerns temporality. No study has followed individuals with repeated dietary assessment to determine whether a change in antioxidant intake precedes a change in CKM stage, so it is not known whether the gradient observed across stages reflects diet acting on disease, disease acting on diet, or shared determinants of both. The second concerns the generalizability of the threshold. No inflection point has been estimated for a CDAI–CKM association in any population other than ours, and whether a comparable plateau exists where dietary distributions differ, or whether its location varies by sex or by race and ethnicity, is unknown. The third concerns the composition of the index. The CDAI weights its 6 components equally, yet these nutrients are not absorbed or metabolized independently, and neither the relative contribution of individual components nor the joint and potentially antagonistic effects among them has been characterized for any CKM endpoint. The fourth is interventional. Trials of high-dose single-nutrient antioxidants in general adult populations and in CKD have been null or unfavorable,^[46–48]^ but no trial has evaluated whether increasing the diversity of food-derived antioxidants alters outcomes in adults in CKM stages 3 and 4, which is the strategy these findings point toward.

### 4.4. Future directions

Three lines of research would be required before these findings could inform clinical decisions. First, prospective cohorts with repeated dietary assessment, ideally using multiple 24-hour recalls or usual intake methods to reduce measurement error, should test whether within-person increases in CDAI precede stabilization or regression of CKM stage. Second, the inflection point should be reestimated in independent cohorts with different dietary distributions before any intake target is proposed, with sex-specific and race and ethnicity-specific thresholds estimated in the same step. Third, and most directly, a randomized trial comparing a whole-food, antioxidant-diverse dietary pattern with usual care in adults in CKM stages 3 and 4 would test the strategy these findings support; no such trial has been conducted. Alongside these, analyses that decompose the composite signal of the CDAI would clarify which components carry the association: supervised learning with permutation importance and Shapley additive explanation values can rank component contributions, and mixture methods such as Bayesian kernel machine regression or latent class analysis can identify intake phenotypes and joint effects.^[49–51]^ Such analyses should account for the NHANES survey design and weights and should be validated through cross-validation and external replication.

## 5. Conclusion

In this cross-sectional analysis of 23,968 U.S. adults, a higher food-derived CDAI was associated with a lower prevalence of advanced CKM syndrome. Compared with Q1, Q2 to Q4 had 13% to 21% lower odds of advanced CKM after full adjustment, with a significant trend across quartiles, and the estimates were consistent in direction after multiple imputation, propensity score matching, and survey-weighted analysis. The association was L-shaped: below a CDAI of approximately 0.36, each 1-unit increase was associated with 4% lower odds of advanced CKM, whereas no association was evident above that value. These results identify the low-to-moderate range of food-derived antioxidant intake as the range in which the association is concentrated, and provide no evidence of additional benefit at higher intake. Because the design is cross-sectional and exposure was measured on a single occasion, the findings establish association rather than effect, and prospective and interventional studies are required before an intake target could be recommended.

## Acknowledgments

We thank Huanxian Liu (Department of Neurology, Chinese PLA General Hospital, Beijing, China) and Haoxian Tang (Department of Clinical Medicine, Shantou University Medical College, Shantou, Guangdong, China) for their valuable comments on the study design and manuscript.

## Author contributions

**Conceptualization:** Yubin Wang, Yingxuan Huang.

**Data curation:** Yubin Wang, Yingxuan Huang.

**Formal analysis:** Yubin Wang, Yingxuan Huang, Xinqi Chen, Yingyi Li.

**Methodology:** Yubin Wang, Yingxuan Huang.

**Supervision:** Yubin Wang, Yingxuan Huang, Yisen Huang, Xiaoqiang Liu.

**Software:** Chanchan Lin.

**Writing – original draft:** Yubin Wang, Yingxuan Huang, Chanchan Lin, Xinqi Chen, Yingyi Li.

**Writing – review & editing:** Yisen Huang, Xiaoqiang Liu.








