# Supplementary material for: Association of the composite dietary antioxidant index with advanced cardiovascular–kidney–metabolic syndrome in U.S. adults: A cross-sectional study based on NHANES 2001–2018
Source: Medicine (Baltimore). 2026 Sep 25;105(39):e50849. doi: 10.1097/MD.0000000000050849 (PMC13619232; doi:10.1097/MD.0000000000050849)
Supplement: Supplementary file 1 [file medi-105-e50849-s001.docx]

**Supplementary Table 1 Definitions of CKM Health Stages (Green indicates that all criteria must be met, yellow indicates that meeting any one criterion is sufficient)**

| **CKM health stages** | **Definition** | **Indicators** | **Criterion** | **Condition** |
| --- | --- | --- | --- | --- |
| Stage 0: No CKM health risk factors | Individuals without overweight/obesity, metabolic risk factors (hypertension, hypertriglyceridemia, MetS, diabetes), CKD, or subclinical/clinical CVD | Without overweight/obesity | BMI <25 kg/m^2^ (or <23 kg/m^2^ if Asian ancestry) | All criteria are met |
|  |  | Without abdominal obesity | Waist circumference <88/102 cm in female/male (or if Asian ancestry <80/90 cm in female/male) |  |
|  |  | Without prediabetes/diabetes | Fasting blood glucose <100 mg/dL and HbA1c < 5.7% and without self-reported diagnosis of prediabetes/diabetes, use of insulin, or oral hypoglycemic agents |  |
|  |  | Without hypertension | SBP <130 mmHg and DBP <80 mmHg without self-reported diagnosis of hypertension or use of antihypertensive medications |  |
|  |  | Without hypertriglyceridemia | Triglycerides < 135 mg/dL |  |
|  |  | Without CKD | Low-risk CKD in KDIGO classification according to eGFR and  UACR: UACR < 30 mg/g and eGFR ≥ 60 ml/min/1.73m^2^. |  |
|  |  | Without subclinical/clinical CVD | Predicted 10-year CVD risk < 20% |  |
|  |  |  | Without history of coronary heart failure, coronary heart disease, heart attack, or stroke |  |
| Stage 1: Excess and/or dysfunctional adiposity | Individuals with overweight/obesity, abdominal obesity, or dysfunctional adipose tissue, without the presence of other metabolic risk factors or CKD | With overweight/obesity | BMI ≥25 kg/m^2^ (or ≥23 kg/m^2^ if Asian ancestry) | Any of the three criteria is met |
|  |  | With abdominal obesity | Waist circumference ≥88/102 cm in women/men (or if Asian ancestry, ≥80/90 cm in women/men) |  |
|  |  | Prediabetes | Fasting blood glucose ≥100-124 mg/dL or HbA1c between 5.7% and 6.4% |  |
|  |  | Without hypertension | SBP <130 mmHg and DBP <80 mmHg without self-reported diagnosis of hypertension or use of antihypertensive medications | All criteria are met |
|  |  | Without hypertriglyceridemia | Triglycerides < 135 mg/dL |  |
|  |  | Without CKD | Low-risk CKD in KDIGO classification according to eGFR and  UACR: UACR < 30 mg/g and eGFR ≥ 60 ml/min/1.73m^2^. |  |
|  |  | Without subclinical/clinical CVD | Predicted 10-year CVD risk < 20% |  |
|  |  |  | Without history of coronary heart failure, coronary heart disease, heart attack, or stroke |  |
| Stage 2: Metabolic risk factors and CKD | Individuals with metabolic risk factors (hypertriglyceridemia, hypertension, MetS, diabetes) or CKD | With hypertriglyceridemia | Triglycerides ≥ 135 mg/dL | Any of the five criteria is met |
|  |  | With hypertension | SBP ≥130mm Hg or DBP ≥80mm Hg or self-reported diagnosis of hypertension or use of antihypertensive medication |  |
|  |  | With diabetes | Fasting blood glucose ≥ 125 mg/dL or HbA1c ≥ 6.5% or self-reported diagnosis of diabetes, use of insulin, or oral hypoglycemic agents |  |
|  |  | With MetS | MetS is defined by the presence of ≥3 of the following: (1) waist circumference ≥88 cm for women and ≥102 cm for men (if Asian ancestry, ≥80 cm for women and ≥90 cm for men), (2) high-density cholesterol <40 mg/dL for men and <50 mg/dL for women; (3) triglycerides ≥150 mg/dL; (4) elevated blood pressure (systolic blood pressure ≥130 mm Hg and/or diastolic blood pressure ≥80 mm Hg and/or use of antihypertensive medications); and (5) fasting blood glucose ≥100 mg/dL. |  |
|  |  | With moderate-to-high-risk CKD | Moderate-to-high-risk CKD in KDIGO classification: UACR ≥ 30 mg/g and eGFR ≥ 60 ml/min/1.73m^2^, UACR < 300 mg/g and eGFR 45-59 ml/min/1.73m^2^, or UACR < 30 mg/g and eGFR 30-44 ml/min/1.73m^2^. |  |
|  |  | Without very high-risk CKD | No very high-risk CKD in KDIGO classification: eGFR ≥ 60 ml/min/1.73m^2^, eGFR 45-59 ml/min/1.73m^2^ and UACR <300 mg/g, eGFR30 ml/min/1.73m^2^, eGFR 30-44 ml/min/1.73m^2^ and UACR <30 mg/g | All criteria are met |
|  |  | Without subclinical/clinical CVD | Predicted 10-year CVD risk < 20% |  |
|  |  |  | Without history of coronary heart failure, coronary heart disease, heart attack, or stroke |  |
| Stage 3: Subclinical CVD in CKM | Subclinical CVD in CKM | With very high-risk CKD | Very high-risk CKD in KDIGO classification: eGFR＜30 ml/min/1.73m^2^, eGFR 30-44 ml/min/1.73m^2^ and UACR ≥30 mg/g, or eGFR 45-59 ml/min/1.73m^2^ and UACR ≥300 mg/g | Any of the two criteria is met |
|  |  | With subclinical CVD | Predicted 10-y CVD risk ≥ 20% |  |
|  |  | Without clinical CVD | Without history of coronary heart failure, coronary heart disease, heart attack, or stroke | The criterion is met |
|  |  | With overweight/obesity | BMI ≥25 kg/m^2^ (or ≥23 kg/m^2^ if Asian ancestry) | Any of the eight criteria is met |
|  |  | With abdominal obesity | Waist circumference ≥88/102 cm in women/men (or if Asian ancestry, ≥80/90 cm in women/men) |  |
|  |  | With hypertriglyceridemia | Triglycerides ≥ 135 mg/dL |  |
|  |  | With hypertension | SBP ≥130mm Hg or DBP ≥80mm Hg or self-reported diagnosis of hypertension or use of antihypertensive medication |  |
|  |  | With prediabetes | Fasting blood glucose ≥100-124 mg/dL or HbA1c between 5.7% and 6.4% |  |
|  |  | With diabetes | Fasting blood glucose ≥ 125 mg/dL or HbA1c ≥ 6.5% or self-reported diagnosis of diabetes, use of insulin, or oral hypoglycemic agents |  |
|  |  | With MetS | MetS is defined by the presence of ≥3 of the following: (1) waist circumference ≥88 cm for women and ≥102 cm for men (if Asian ancestry, ≥80 cm for women and ≥90 cm for men), (2) high-density cholesterol <40 mg/dL for men and <50 mg/dL for women; (3) triglycerides ≥150 mg/dL; (4) elevated blood pressure (systolic blood pressure ≥130 mm Hg and/or diastolic blood pressure ≥80 mm Hg and/or use of antihypertensive medications); and (5) fasting blood glucose ≥100 mg/dL. |  |
|  |  | With moderate-to-high-risk CKD | Moderate-to-high-risk CKD in KDIGO classification: UACR ≥ 30 mg/g and eGFR ≥ 60 ml/min/1.73m^2^, UACR < 300 mg/g and eGFR 45-59 ml/min/1.73m^2^, or UACR < 30 mg/g and eGFR 30-44 ml/min/1.73m^2^. |  |
| Stage 4: Clinical CVD in CKM | Clinical CVD (coronary heart disease, heart failure, stroke, peripheral artery disease, AFib) among individuals with excess/dysfunctional adiposity, other metabolic risk factors, or CKD | With clinical CVD | History of coronary heart failure, coronary heart disease, heart attack, or stroke | The criterion is met |
|  |  | With overweight/obesity | BMI ≥25 kg/m^2^ (or ≥23 kg/m^2^ if Asian ancestry) | Any of the nine criteria is met |
|  |  | With abdominal obesity | Waist circumference ≥88/102 cm in women/men (or if Asian ancestry, ≥80/90 cm in women/men) |  |
|  |  | With hypertriglyceridemia | Triglycerides ≥ 135 mg/dL |  |
|  |  | With hypertension | SBP ≥130mm Hg or DBP ≥80mm Hg or self-reported diagnosis of hypertension or use of antihypertensive medication |  |
|  |  | With prediabetes | Fasting blood glucose ≥100-124 mg/dL or HbA1c between 5.7% and 6.4% |  |
|  |  | With diabetes | Fasting blood glucose ≥ 125 mg/dL or HbA1c ≥ 6.5% or self-reported diagnosis of diabetes, use of insulin, or oral hypoglycemic agents |  |
|  |  | With MetS | MetS is defined by the presence of ≥3 of the following: (1) waist circumference ≥88 cm for women and ≥102 cm for men (if Asian ancestry, ≥80 cm for women and ≥90 cm for men), (2) high-density cholesterol <40 mg/dL for men and <50 mg/dL for women; (3) triglycerides ≥150 mg/dL; (4) elevated blood pressure (systolic blood pressure ≥130 mm Hg and/or diastolic blood pressure ≥80 mm Hg and/or use of antihypertensive medications); and (5) fasting blood glucose ≥100 mg/dL. |  |
|  |  | With moderate-to-high-risk CKD | Moderate-to-high-risk CKD in KDIGO classification: UACR ≥ 30 mg/g and eGFR ≥ 60 ml/min/1.73m^2^, UACR < 300 mg/g and eGFR 45-59 ml/min/1.73m^2^, or UACR < 30 mg/g and eGFR 30-44 ml/min/1.73m^2^. |  |
|  |  | With very high-risk CKD | Very high-risk CKD in KDIGO classification: eGFR＜30 ml/min/1.73m^2^, eGFR 30-44 ml/min/1.73m^2^ and UACR ≥30 mg/g, or eGFR 45-59 ml/min/1.73m^2^ and UACR ≥300 mg/g |  |

Abbreviations: BMI, body mass index; CKD, chronic kidney disease; CKM, cardiovascular-kidney-metabolic; CVD, cardiovascular disease; DBP, diastolic blood pressure; eGFR, estimated glomerular filtration rate; MetS, metabolic syndrome; HDL, high-density lipoprotein; KDIGO, The Kidney Disease, Improving Global Outcomes; SBP, systolic blood pressure; UACR, urinary albumin to creatinine ratio.

**References:**

1. Ndumele CE, Rangaswami J, Chow SL, et al. Cardiovascular-Kidney-Metabolic Health: A Presidential Advisory From the American Heart Association [published correction appears in Circulation. 2024 Mar 26;149(13):e1023. doi: 10.1161/CIR.0000000000001241]. Circulation. 2023;148(20):1606-1635. doi:10.1161/CIR.0000000000001184
