# Supplementary material for: Association of the composite dietary antioxidant index with advanced cardiovascular–kidney–metabolic syndrome in U.S. adults: A cross-sectional study based on NHANES 2001–2018
Source: Medicine (Baltimore). 2026 Sep 25;105(39):e50849. doi: 10.1097/MD.0000000000050849 (PMC13619232; doi:10.1097/MD.0000000000050849)
Supplement: Supplementary file 2 [file medi-105-e50849-s002.docx]

**Supplementary Table 2 Association between CDAI and advanced CKM syndrome after multiple imputations of missing data**

|  | **Crude model** | |  | **Model 1** | |  | **Model 2** | |
| --- | --- | --- | --- | --- | --- | --- | --- | --- |
|  | **OR (95%CI)** | ***P* value** |  | **OR (95%CI)** | ***P* value** |  | **OR (95%CI)** | ***P* value** |
| CDAI quartile |  |  |  |  |  |  |  |  |
| Q1 | Reference |  |  | Reference |  |  | Reference |  |
| Q2 | 0.81 (0.75~0.87) | <0.001 |  | 0.74 (0.68~0.81) | <0.001 |  | 0.84 (0.76~0.92) | <0.001 |
| Q3 | 0.63 (0.59~0.68) | <0.001 |  | 0.60 (0.54~0.66) | <0.001 |  | 0.73 (0.66~0.81) | <0.001 |
| Q4 | 0.51 (0.47~0.55) | <0.001 |  | 0.59 (0.53~0.65) | <0.001 |  | 0.77 (0.69~0.85) | <0.001 |
| *P* for trend |  | <0.001 |  |  | <0.001 |  |  | <0.001 |

Abbreviations: CDAI, composite dietary antioxidant index; CKM, cardiovascular kidney metabolic; PIR, poverty income ratio.

The crude model was not adjusted for any covariates. Model 1 was adjusted for age and sex. Model 2 was further adjusted for race, marital status, PIR, education level, body mass index, total energy intake, smoking status, alcohol intake, physical activity.
