# Supplementary material for: Association of the composite dietary antioxidant index with advanced cardiovascular–kidney–metabolic syndrome in U.S. adults: A cross-sectional study based on NHANES 2001–2018
Source: Medicine (Baltimore). 2026 Sep 25;105(39):e50849. doi: 10.1097/MD.0000000000050849 (PMC13619232; doi:10.1097/MD.0000000000050849)
Supplement: Supplementary file 3 [file medi-105-e50849-s003.docx]

**Supplementary Table 3 Association between CDAI and advanced CKM syndrome after propensity score matching**

|  | **Crude model** | |  | **Model 1** | |  | **Model 2** | |
| --- | --- | --- | --- | --- | --- | --- | --- | --- |
|  | **OR (95%CI)** | ***P* value** |  | **OR (95%CI)** | ***P* value** |  | **OR (95%CI)** | ***P* value** |
| CDAI quartile |  |  |  |  |  |  |  |  |
| Q1 | Reference |  |  | Reference |  |  | Reference |  |
| Q2 | 0.94 (0.83~1.07) | 0.375 |  | 0.94 (0.82~1.07) | 0.335 |  | 0.93 (0.81~1.07) | 0.312 |
| Q3 | 0.87 (0.76~0.99) | 0.035 |  | 0.86 (0.76~0.99) | 0.030 |  | 0.85 (0.74~0.99) | 0.034 |
| Q4 | 0.88 (0.77~1.01) | 0.069 |  | 0.88 (0.77~1.01) | 0.068 |  | 0.86 (0.73~1.02) | 0.086 |
| *P* for trend |  | 0.030 |  |  | 0.030 |  |  | 0.045 |

Abbreviations: CDAI, composite dietary antioxidant index; CKM, cardiovascular kidney metabolic; PIR, poverty income ratio.

The crude model was not adjusted for any covariates. Model 1 was adjusted for age and sex. Model 2 was further adjusted for race, marital status, PIR, education level, body mass index, total energy intake, smoking status, alcohol intake, physical activity.
