# Supplementary material for: Association of the composite dietary antioxidant index with advanced cardiovascular–kidney–metabolic syndrome in U.S. adults: A cross-sectional study based on NHANES 2001–2018
Source: Medicine (Baltimore). 2026 Sep 25;105(39):e50849. doi: 10.1097/MD.0000000000050849 (PMC13619232; doi:10.1097/MD.0000000000050849)
Supplement: Supplementary file 4 [file medi-105-e50849-s004.docx]

**Supplementary Table 4 Weighted analysis of the Association between CDAI and advanced CKM syndrome**

|  | **Crude model** | |  | **Model 1** | |  | **Model 2** | |
| --- | --- | --- | --- | --- | --- | --- | --- | --- |
|  | **OR (95%CI)** | ***P* value** |  | **OR (95%CI)** | ***P* value** |  | **OR (95%CI)** | ***P* value** |
| Continuous CDAI | 0.94 (0.93~0.96) | <0.001 |  | 0.95 (0.93~0.97) | <0.001 |  | 1 (0.98~1.02) | 0.682 |
| CDAI quartile |  |  |  |  |  |  |  |  |
| Q1 | Reference |  |  | Reference |  |  | Reference |  |
| Q2 | 0.82 (0.74~ 0.91) | <0.001 |  | 0.71 (0.61~ 0.82) | <0.001 |  | 0.88 (0.76~1.02) | 0.092 |
| Q3 | 0.67 (0.61~0.74) | <0.001 |  | 0.58 (0.51~ 0.68) | <0.001 |  | 0.84 (0.71~0.99) | 0.036 |
| Q4 | 0.53 (0.48~0.60) | <0.001 |  | 0.52 (0.44~0.61) | <0.001 |  | 0.83 (0.69~0.99) | 0.038 |
| *P* for trend |  | <0.001 |  |  | <0.001 |  |  | 0.039 |

Abbreviations: CDAI, composite dietary antioxidant index; CKM, cardiovascular kidney metabolic.

Crude model was not adjusted for any covariates. Model 1 was adjusted for age and sex. Model 2 was further adjusted for race, marital status, poverty-income ratio, education level, body mass index, total energy intake, smoking status, alcohol intake, physical activity.
